# Characterization of Porous TiO_2_ Surfaces Formed on 316L Stainless Steel by Plasma Electrolytic Oxidation for Stent Applications

**DOI:** 10.3390/jfb3020349

**Published:** 2011-05-11

**Authors:** Zhiguang Huan, Lidy E. Fratila-Apachitei, Iulian Apachitei, Jurek Duszczyk

**Affiliations:** Department of BioMechanical Engineering, Delft University of Technology, Mekelweg 2, Delft 2628 CD, The Netherlands; Email: z.huan@tudelft.nl (Z.H.); i.apachitei@tudelft.nl (I.A.); j.duszczyk@tudelft.nl (J.D.)

**Keywords:** drug eluting stent, plasma electrolytic oxidation, titanium oxide layer, stainless steel, surface porosity

## Abstract

In this study, a porous oxide layer was formed on the surface of 316L stainless steel (SS) by combining Ti magnetron sputtering and plasma electrolytic oxidation (PEO) with the aim to produce a polymer-free drug carrier for drug eluting stent (DES) applications. The oxidation was performed galvanostatically in Na_3_PO_4_ electrolyte. The surface porosity, average pore size and roughness varied with PEO treatment duration, and under optimum conditions, the surface showed a porosity of 7.43%, an average pore size of 0.44 µm and a roughness (Ra) of 0.34 µm. The EDS analyses revealed that the porous layer consisted of Ti, O and P. The cross-sectional morphology evidenced a double-layer structure, with a porous titania surface and an un-oxidized dense Ti film towards the interface with 316L SS. After the PEO treatment, wettability and surface free energy increased significantly. The results of the present study confirm the feasibility of forming a porous TiO_2_ layer on stainless steel by combining sputtering technology and PEO. Further, the resultant porous oxide layer has the potential to be used as a drug carrier for DES, thus avoiding the complications associated with the polymer based carriers.

## 1. Introduction

In recent years, the combination of stents, able to inhibit recoil and negative tissue remodeling with drugs that inhibit neointimal hyperplasia has emerged as a highly promising alternative to reduce in-stent restenosis in the treatment of atherosclerosis [1,2]. These drug eluting stents (DES) consist mostly of a metallic scaffold and a polymer coating which contains drugs. As the drugs are released from the coating after implantation, the rates of restenosis are substantially reduced by inhibition of cells’ proliferation, as revealed by numerous large clinical trials [3,4]. Despite the advantages over bare metal stents, the incidence of late stent thrombosis and the development of late restenosis have raised issues about the long-term safety and efficacy of DES [5,6]. Both late occurring complications have been related to the characteristics of the polymer matrix, which can cause a marked inflammatory response leading to incomplete re-endothelialization and neointimal proliferation after completion of drug release [7]. 

To avoid the complications associated with polymer-based DES, development of polymer-free drug-eluting stents is desirable. From a biomedical point of view, titanium oxide (TiO_2_) surfaces with their excellent biochemical stability and blood compatibility can be a promising alternative to polymer matrices [8,9,10]. Therefore, it is suggested that a TiO_2_ layer can protect a metallic stent from direct contact with the vessel wall after drug elution is completed. 

In this study, porous TiO_2_ layers have been produced on a 316L stainless steel substrate, which is the most commonly used material for cardiovascular stents, by sputtering a titanium film on the substrate that was subsequently oxidized by plasma electrolytic oxidation (PEO). Deposition of a valve metal on steel by different methods followed by PEO has been previously used for formation of protective coatings [11,12]. Plasma electrolytic oxidation is an electrochemical method used to produce porous oxide layers on valve metals and their alloys [13,14]. The process occurs at high voltages (above the breakdown voltages) and the characteristics of the porous layers may be controlled by adjusting the process parameters. The process has been applied to enhance surface biofunctionality of titanium alloys used in orthopedic and dental implants [15,16], while its application for the fabrication of DES is rare. Therefore, the purpose of the current study was to evaluate the feasibility of PEO to produce porous polymer-free drug carriers for DES.

## 2. Experimental Section

### 2.1. Sample Preparation and PEO Treatment

Samples of 8 cm × 1 cm × 0.1 cm were cut from a 316L stainless steel (SS) sheet and were successively cleaned in acetone, ethanol and deionized water for 10 min each. Then the specimens were coated with a Ti film of 5 µm thickness by magnetron sputtering. The as-coated samples are denoted as 5-Ti-SS. 

Prior to the PEO treatment, the as-coated samples were ultrasonically cleaned in acetone and deionized water. PEO was carried out in a double-wall glass electrolytic cell with a volume of 800 mL. The samples were screwed to an insulated metallic rod and suspended in the centre of the electrolytic cell as anode, surrounded by a cylindrical steel cathode. As an electrolyte, a solution of 0.04 M tri-sodium phosphate (Na_3_PO_4_) was used that was cooled during the process by circulation of cooling water (10 °C) through the electrolytic cell jacket using an external thermostat. Agitation of the electrolyte was maintained at a speed of 500 rpm using a magnetic stirrer (Ika, NL). PEO was performed under galvanostatic conditions at a current density of 5 A/dm^2^. During the oxidation process, the voltage was automatically recorded, and the oxidation time was up to 40 min to get an overview of the relationship between the oxidation time and the PEO response. After selected durations, the process was stopped and the resultant samples were thoroughly cleaned with deionized water, dried using blowing air and stored in desiccator until further testing.

### 2.2. Surface Characterization

After sputter coating for enhanced conductivity, the surface morphology of the oxidized samples was examined by scanning electron microscopy (SEM, JSM-6500F, JEOL) using an accelerating voltage of 5 kV. The elemental composition was estimated on the surface and cross-section by an energy dispersive X-ray spectrometer (EDS, INCA Energy, Oxford Instruments) coupled with the SEM equipment. To observe the 316L/Ti and Ti/TiO_2_ interfaces, the cross section images of the specimens were also investigated. Further, the oxide layer’s thickness was measured directly from the cross section images of the specimens. A Taylor-Hobson Surtronic 3^+^ surface texture-meter was used to determine the average surface roughness (Ra) of the samples. Ten random measurements were taken for each sample followed by statistical analysis to determine the mean Ra value. Pore’s diameter was measured from SEM images using the Photoshop^®^ software based on which surface porosity was estimated.

The dynamic advancing contact angles were determined with a Krüss DSA 100 drop shape analyzer using deionized water and diiodomethane. A volume of 10 µL liquid was placed automatically on the tested surface using a microlitre syringe. Upon contact with the surface the increasing droplet profile was measured at 1 s intervals for 33 s. For every sample, triplicate measurements were performed in the two different wetting liquids. Surface free energy was calculated according to Fowkes’ theory. The values reported represent the average and standard deviations for contact angles in water and total surface free energy.

### 2.3. Statistics

The experimental values were analyzed using the Student’s t-test and are expressed as the mean values ± standard deviation (SD). A *p*-value < 0.05 was considered statistically significant.

## 3. Results and Discussion

### 3.1. Voltage-Time Responses during the PEO Process

**Figure 1:**
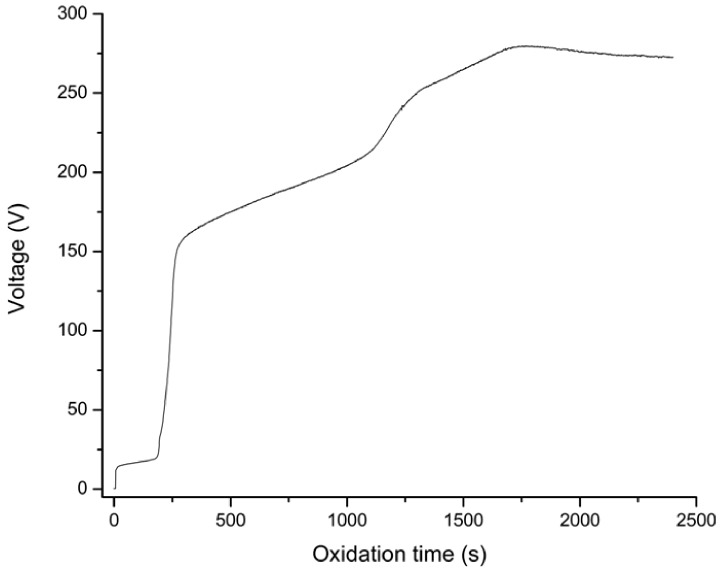
Voltage transients during plasma electrolytic oxidation (PEO) of 5-Ti-SS samples in Na_3_PO_4_ electrolyte at a current density of 5 A/dm^2^.

The evolution of voltage with the PEO treatment time is shown in Figure 1. During the initial 180 s of PEO treatment, the voltage-time responses revealed a slow voltage rise probably due to preferential initial oxide growth at the fine flaws on the sputtered surface representing the easiest path for the current flaw. The voltage then rose rapidly to about 160 V indicating formation of a dielectric barrier layer with high electric resistance. As the PEO treatment continued the voltage increased further, however with a reduced slope. When the anodic voltage reached a value of *ca*. 220 V, large numbers of small sparks were observed to move rapidly and randomly across the surface of the oxide film indicative of dielectric breakdown due to impact ionisation [14,17,18]. The size of the sparks increased with prolonged PEO treatment, while their density and moving speed decreased so that at about 250 V they became almost immobile. The maximum voltage reached was 280 V after which it started to slowly decrease indicating that layer growth could not be sustained anymore. Apart from the initial region of low slope, the voltage-time response followed the general trend found when bulk titanium substrates are PEO treated in phosphate-based solutions [17,19]. 

It is known that under galvanostatic conditions, the characteristics of the porous layers can vary with PEO treatment duration as a function of sparks morphology, density, mobility and intensity [14,20,21], and the gas released through the locally softened material [17]. It is therefore of interest to evaluate layer properties at different voltages during the PEO process. In this study, the evolution of surface morphology was assessed at 220 V, 250 V and 280 V, and the focus was on porosity, pore size and pore density, which are considered important for the potential application as drug carriers for drug eluting stents.

### 3.2. Surface Morphology and Chemical Composition of the PEO Layers

**Figure 2:**
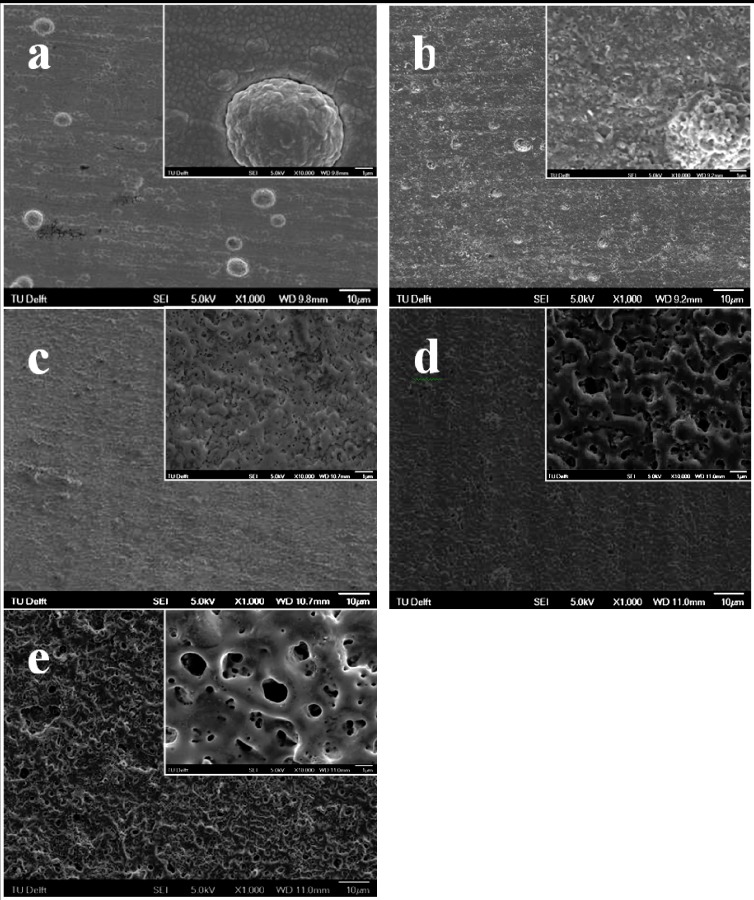
Surface morphology of 5-Ti-SS samples at different stages during the PEO process: (**a**) before PEO; (**b**) PEO to 220 V; (**c**) PEO to 250 V and (**d**) PEO to 280 V. The insets represent the treated surfaces at enhanced magnification.

The surface morphology of the layers formed on the titanium sputtered stainless steel substrate during the PEO at the three different voltages selected is shown in Figure 2. The stainless steel was completely covered by a dense Ti layer showing fine grain size and some larger agglomerates distributed homogeneously on the surface (Figure 2a). The surface morphology of the sample at 200 V is shown in Figure 2b as representative for the PEO stage between 150 and 220 V. It can be seen that very tiny pores appeared on the surface, while the pore density was quite low. The Ti agglomerates as observed on the surfaces before PEO were still visible but their surface turned porous as a result of the surface treatment. At this stage, generation of gas from the surface without visible sparks was observed during the PEO process, which resulted in the formation of tiny pores on the surface. At the beginning of sparking (220 V), very fine pores of less than 200 nm (Table 1) were uniformly distributed on the surface (Figure 2c) associated with formation of the very small and mobile sparks. Further, the Ti agglomerates observed on the surface before PEO were not visible anymore indicating that they underwent oxidation during the process with formation of a porous structure that merged into the rest of the layer. At 250 V (Figure 2d), the surface revealed larger pores, a lower pore density (Table 1) and a rougher surface that may be attributed to the enhanced discharging energy with increasing voltage causing fewer but more intense moving sparks on the surface. As the voltage further increased to the maximum value of 280 V, a typical PEO microstructure developed (Figure 2e) with few large pores of 1–2 µm surrounded by smaller (<1 µm) ones in a crater-like morphology. The evolution of surface morphology observed during the PEO of the titanium film is quite similar to that of bulk Ti and its alloys [21,22]. 

**Table 1:** Surface porosity, average pore size and pore density of 5-Ti-SS at different stages during the plasma electrolytic oxidation (PEO) process as indicated by the final voltage.

Voltage during PEO	220 V	250 V	280 V
Surface porosity (%)	3.18	7.43	7.89
Average pore size (µm)	0.13	0.44	0.57
Pore density (no. pores/mm^2^)	2.49 × 10^6^	5.10 × 10^5^	2.97 × 10^5^

The surface porosity, average pore size and pore density corresponding to the three different stages of PEO treatment are shown in Table 1. The results indicated that surface porosity and average pore size increased with increasing voltage while pore density decreased. The surface obtained at 280 V presented the highest porosity and pore size whereas the pore density was the lowest and the pore size distribution rather broad. Further, it was noticed that the porosity at 280 V was just slightly higher than that at 250 V. 

**Figure 3:**
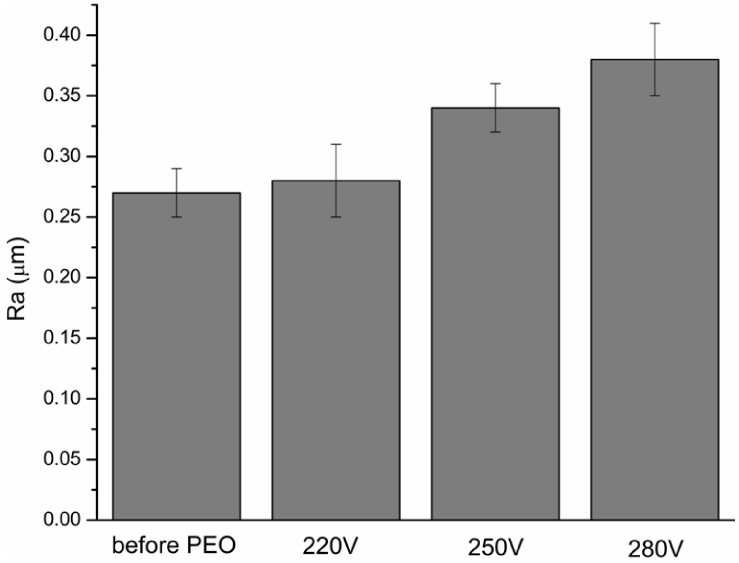
Development of average surface roughness (Ra) during the PEO process of 5-Ti-SS samples in Na_3_PO_4_ electrolyte at a current density of 5 A/dm^2^.

The surface roughness of the specimens before and after PEO treatment is shown in Figure 3. As compared to the original 5-Ti-SS surface (Ra = 0.27 ± 0.02 µm), the oxidized specimens showed higher surface roughness regardless of the voltage at which the PEO process was finished. The increase in roughness is expected due to localized growth of the layer at the breakdown sites, formation of pores and increased layer thickness [23,24]. Based on the morphological characterization of the PEO treated surfaces at the three different voltages, it is supposed that the porous surface obtained at 250 V is the most suitable candidate as drug carrier because it showed a relatively high surface porosity combined with a more uniform pore size distribution and an intermediate average roughness.

**Figure 4:**
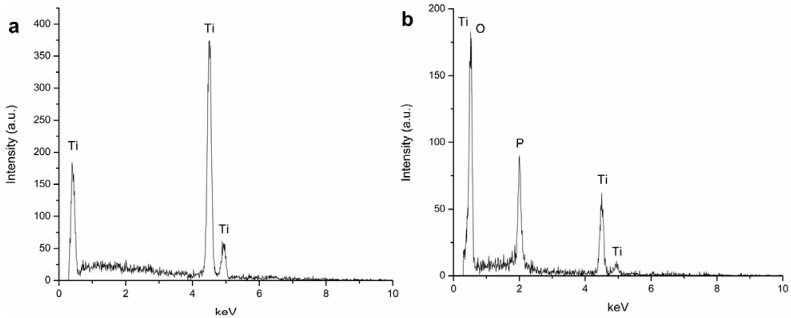
Elemental composition of 5-Ti-SS surfaces: (**a**) before PEO treatment and (**b**) after PEO treatment to 250 V.

The elemental composition of the Ti film before PEO treatment and of the porous surface after PEO treatment at 250 V was assessed by energy dispersive X-ray spectrometry (EDS) analyses (Figure 4). Before the PEO treatment, the surface is exclusively composed of Ti, and no element from the stainless steel substrate was detected (Figure 4a). In comparison, the oxidized surface (Figure 4b) showed the presence of O in large amounts (65.04 at[e1] %) followed by Ti and P (24.71 at% and 10.25 at%, respectively). The presence of P is due to the incorporation of phosphate from the electrolyte during the PEO process. No element from the stainless steel substrate was present on the treated surface. According to previous studies, both crystalline (anatase, rutile) and amorphous titanium oxide structures are found by conversion of the metallic substrate during PEO of titanium in sodium phosphate electrolyte [25]. Considering the similarity between voltage-time responses during PEO processes on Ti bulk and film, it is reasonable to believe that the porous oxide layers formed on the stainless steel substrate consist of both crystalline and amorphous titanium oxide. 

### 2.3. Morphology and Elemental Distribution across Oxide Layers Thickness

**Figure 5:**
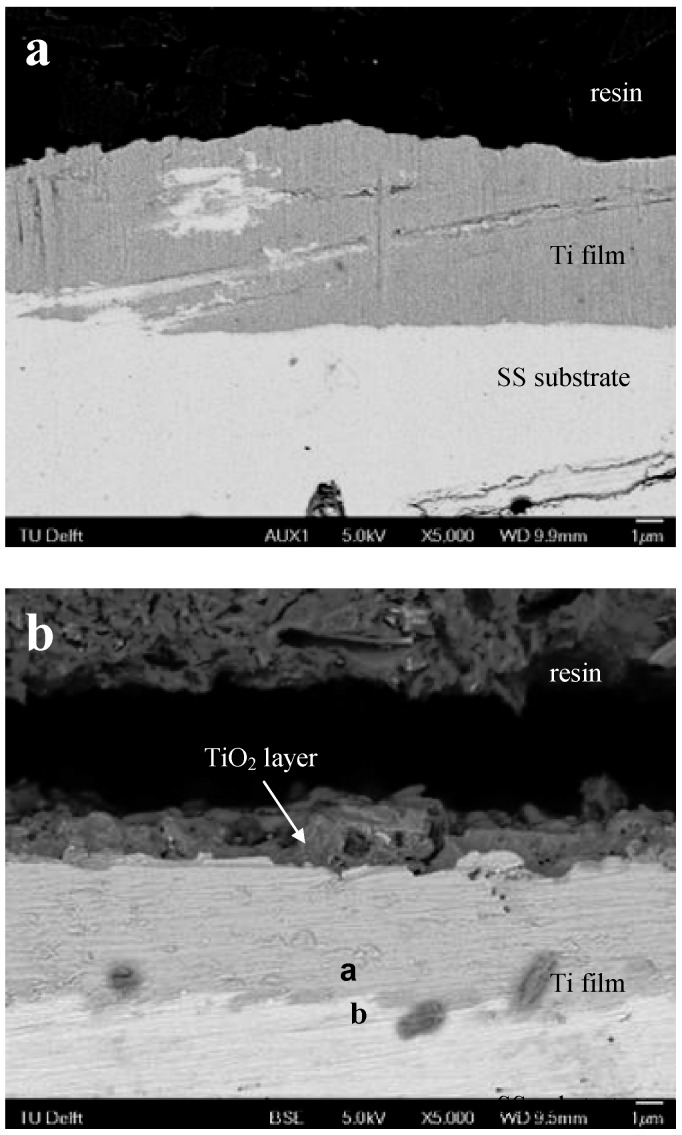
Cross-section morphology of 5-Ti-SS samples: (**a**) before and (**b**) after PEO treatment at 250 V, as observed by back-scattered SEM.

**Figure 6:**
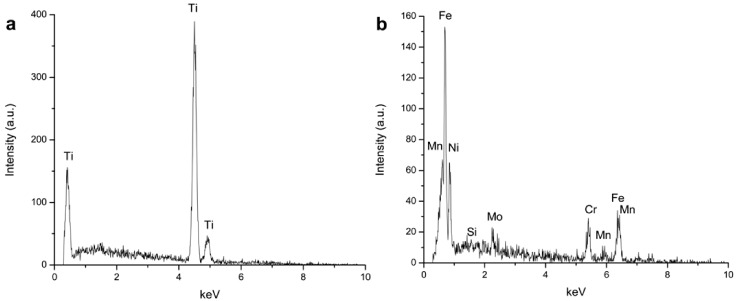
Elemental composition of the cross-section after PEO treatment at 250 V: (**a**) Ti film and (**b**) stainless steel (SS) substrate. The EDS analyses were performed at location a (for Ti) and b (for SS) indicated in Figure 5b.

The polished cross section of the Ti-sputtered stainless steel specimen before and after PEO treatment at 250 V was observed by SEM and the results are presented in Figure 5. The Ti sputtered film (Figure 5a) showed a good thickness uniformity and no gaps were detected at the interface with the stainless steel substrate indicating a good adhesion of the film. After PEO treatment (Figure 5b), part of the Ti was converted to TiO_2_ forming an oxide layer with a thickness of *ca*.1.5 µm on the surface. Furthermore, EDS measurements have been conducted at the SS/Ti interface after the PEO treatment (see locations a and b in Figure 5b) to investigate if there was any diffusion between the two materials during the PEO process and/or sputtering. The results (Figure 6) showed only elemental Ti in the un-oxidized Ti (location a) whereas no detectable Ti was found in the stainless steel substrate (location b). It is therefore believed that diffusion of atoms between the Ti film and stainless steel substrate, if happens, would not be significant.

According to previous reports, both the magnetron sputtering and PEO process render layers with good adhesion strength. For example, Ding *et al*. found that a Ti coating formed by magnetron sputtering shows an average bonding strength higher than 80 MPa [26]. Meanwhile, according to the report from Huang *et al*., the bonding strength between a titania coating and pure Ti substrate is more than 40 MPa when the applied voltage in the PEO process is 250 V [27]. It is therefore reasonable to expect that, with the absence of gaps along their interfaces, the titanium oxide surface layer, un-oxidized Ti film and stainless steel substrate could possess good bonding strength between each other, possibly avoiding the peel-off that is always a concern with polymer coating [28,29]. Besides, the combination of dense Ti and porous TiO_2_ as proposed in the present study may add extra benefit by acting as an effective barrier against ionic release from stainless steel substrate, closely associated with allergy and in-stent restenosis, since an oxide layer on SS, either native or electrochemically formed, is not likely to be an efficient protecting layer against ionic release in the long-term [30]. 

### 2.4. Wettability and Surface Free Energy

**Figure 7:**
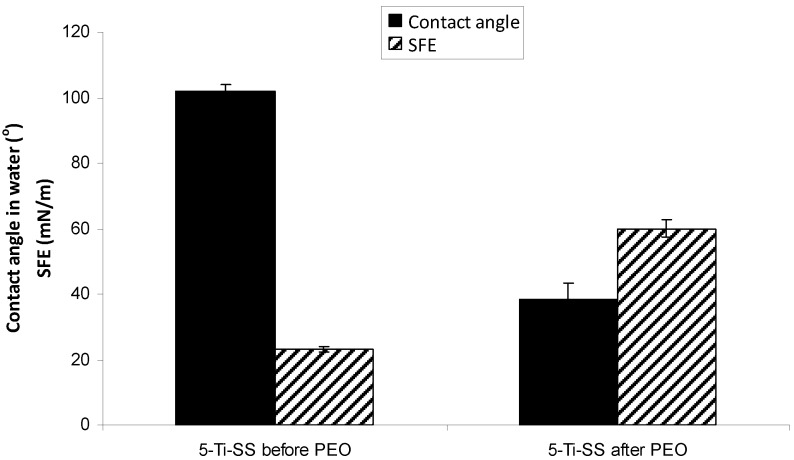
Water contact angle and total surface free energy (SFE) of 5-Ti-SS samples before and after PEO treatment.

Figure 7 includes the results of contact angle measurements and as-calculated surface free energy (SFE) in water before and after PEO treatment for the 5-Ti-SS samples. The PEO-treated sample at 250 V was used as representative. PEO treatment showed a significant influence on the wettability of the surfaces. Deionized water on the untreated 5-Ti-SS samples formed a regular drop, with a contact angle of about 102.0°. After PEO treatment, the contact angle decreased to around 38.2°, indicating a change from hydrophobic to hydrophilic state. The total SFE of the PEO layers was significantly higher than that of the original Ti film. The marked lowering of contact angle and higher SFE relative to the untreated surfaces may be determined by the transition from metal to oxide structures and the increased low scale roughness and porosity [31,32].

It is well known that platelet activation, which plays a key role in thrombogenicity of blood contacting materials, depends on surface properties of the material such as surface charge, wettability, surface free energy, roughness, balance between hydrophobic and hydrophilic groups, and presence of chemical groups on the surface [33,34]. Song SJ *et al*. [29] found that the numbers of platelets that adhere on the surface can be significantly decreased by depositing a TiO_2_ film on stainless steel, indicating that TiO_2_ films have much better compatibility. Meanwhile, the research from Wang GX *et al.* [35] confirmed that a TiO_2_-coated NiTi intravascular stent showed increased surface hydrophilicity and enhanced anticoagulation properties. Further, it has been pointed out that the combination of a TiO_2_ coating and specific drugs further enhance the surface blood compatibility and anticoagulation properties [29,36]. 

It is clear from these results that a porous TiO_2_ layer with a good adhesion to the substrate can be produced on the surface of medical stainless steel by combining magnetron sputtering and PEO process. The properties of the layers such as surface porosity and average pore size can be adjusted by changing the process parameters. Such a TiO_2_ porous layer may provide improved drug loading ability which makes it a good candidate as drug carrier. Once the anti-restenotic drug is released, the TiO_2_ layer would continue acting as a blood compatible barrier between the stainless steel and blood. To confirm this hypothesis, follow-up research should focus on evaluation of the drug-release kinetics and blood compatibility of the layer. 

## 4. Conclusions

In this study, a porous oxide layer was formed on the surface of 316L stainless steel by combining Ti magnetron sputtering and plasma electrolytic oxidation (PEO) process with the aim to produce a polymer-free drug carrier for drug eluting stent (DES) applications. The morphology of the resultant layers, their elemental composition as well as the wettability and surface free energy has been examined. It was found that layer properties, such as surface porosity, pore size and roughness could be changed by adjusting the duration of the PEO treatment with an optimal condition found after about 23 min (final voltage 250 V). The EDS analyses revealed the presence of O, Ti and P on the oxidized surfaces, indicating that the layer consisted of TiO_2_ with P incorporation from the electrolyte. The cross-sectional morphology revealed defect-free interfaces between the SS substrate, un-oxidized Ti and TiO_2_ surface layer. In addition, the wettability and surface free energy of the oxidized samples were significantly higher than those of the Ti sputtered SS. The findings of this study suggest that a porous TiO_2_ layer can be formed on stainless steel by combining sputtering technology and PEO. Further, the resultant oxide layer has the potential to be used as a drug carrier for DES, thus avoiding the complications associated with the polymer based carriers.
